# The Efficacy of an Optimized, Low-Intensity Photodynamic Therapy Protocol with 10% 5-ALA Nanoemulsion in Refractory Vulvar Lichen Sclerosus: Impact on Quality of Life and Sexual Function

**DOI:** 10.3390/jcm15083155

**Published:** 2026-04-21

**Authors:** Katarzyna Beutler, Alina Jankowska-Konsur, Danuta Nowicka

**Affiliations:** 1 University Centre of General Dermatology and Oncodermatology, Wroclaw Medical University, 50-556 Wroclaw, Polandalina.jankowska-konsur@umw.edu.pl (A.J.-K.); 2 Division of Aesthetic Dermatology and Regenerative Medicine of the Skin, Wroclaw Medical University, 50-368 Wroclaw, Poland

**Keywords:** photodynamic therapy, vulvar lichen sclerosus, female sexual function, patient-reported outcomes

## Abstract

**Background:** Treatment options for vulvar lichen sclerosus (VLS) remain limited; therefore, therapies that improve quality of life and reduce neoplastic risk are needed. Photodynamic therapy (PDT) is a potential option. This study aimed to evaluate quality of life and sexual function in patients treated according to the protocol used at our institution. **Methods:** Forty patients with refractory VLS underwent PDT using a 10% 5-aminolevulinic acid nanoemulsion (Ameluz^®^) applied to lesions under an occlusive aluminum foil dressing. Patients received 1–6 sessions of 10 min illumination (LED: 37 J/cm^2^, ~77 mW/cm^2^) at 4–6-week intervals. The Dermatology Life Quality Index (DLQI) and Female Sexual Function Index (FSFI) were used for assessment. **Results:** Thirty-seven participants answered DLQI, while 20 declared themselves to be sexually active and were included in the analysis. Greater number of PDT sessions was associated with a lower DLQI score (τ = −0.583; adjusted *p* < 0.001). The number of PDT sessions and the total FSFI score (*p* = 0.014), as well as desire (*p* = 0.016), arousal (*p* = 0.020), orgasm (*p* = 0.020), and satisfaction (*p* = 0.016) domains were significantly correlated. Age correlated positively with DLQI scores (*p* = 0.016), indicating greater disease burden in older patients. Longer disease duration was also associated with poorer quality of life (*p* = 0.020). **Conclusions:** PDT can be considered an effective treatment for patients with VLS refractory to standard topical corticosteroid and calcineurin inhibitor therapies when delivered using a refined, patient-centered protocol. This optimized approach used in our institution is based on short irradiation time and precise light delivery, providing a favorable balance between therapeutic efficacy, patient comfort, and treatment feasibility. Our findings also suggest that the cumulative number of PDT sessions is a key factor for clinical response. Further studies should address long-term outcomes.

## 1. Introduction

Vulvar lichen sclerosus (VLS) is a chronic inflammatory disease whose etiology is not fully established. It most commonly occurs in postmenopausal women but can occur in people of any age. Lesions mainly occur in the anogenital area, which includes the perineum, vulva, and often the perianal area. The typical clinical picture consists of porcelain-white, atrophic patches, often with purpura, fissures or erosions. The most common symptoms reported by patients include itching, burning, pain, dyspareunia and discomfort. In advanced cases, scarring, atrophy of the labia minora, narrowing of the vaginal entrance, and problems with sexual function and urination may occur. Although the disease may not cause any symptoms, lack of treatment causes permanent changes in the body and increases the likelihood of developing vulvar squamous cell carcinoma (2 to 6% risk) during a woman’s lifetime [1,2,3,4,5,6]. Although vulvar lichen sclerosus has no universally accepted formal staging system, disease severity may be assessed clinically using validated scoring tools such as the Clinical Scoring System in Vulvar Lichen Sclerosus (CSS VLS), which classifies findings as grade 1 (moderate) or grade 2 (severe) [7].

The etiology of lichen sclerosus of the vulva is not fully understood. It is currently known that autoimmune mechanisms, genetic predisposition and disorders in the functioning of keratinocytes and the skin microenvironment play an important role. The immune response is dominated by a Th1 response, with elevated levels of pro-inflammatory cytokines such as IFN-γ, CXCL9 and CXCL10, and increased lymphocytic infiltration, mainly CD4+, CD8+ and FOXP3+ T cells. The concentration of microRNA-155, which regulates the immune response and promotes chronic inflammation, is increased and is also important. Keratinocytes show signs of cellular stress, necroptosis, and impaired proliferation and differentiation, resulting in epithelial atrophy and fibrosis. In addition, degenerative processes are exacerbated by changes in the expression of genes associated with the cell cycle and reduced basal cell regeneration. Other factors that may contribute to the disease include reduced estrogen levels, trauma, and disturbed intestinal and vaginal microflora [1,8,9,10,11,12]. In most cases, the diagnosis is made on the basis of medical history and physical examination, and a biopsy is performed only in questionable or atypical cases [2].

The quality of life of women suffering from lichen sclerosus significantly decreases [13,14]. The disease also has a negative impact on their sexual functioning. Chronic itching, pain, burning, skin cracks, dyspareunia and anatomical changes in the vulva result in reduced sexual activity, less satisfaction with sex life, a poorer image of one’s own genitals and greater anxiety. More than half of women with lichen sclerosus experience sexual dysfunction, most commonly in the form of pain during intercourse (dyspareunia) and decreased desire, arousal, orgasm and satisfaction [15,16].

The disease also reduces social, professional and mental functioning. Symptoms of depression and withdrawal from sexual and social life are reported by a large proportion of patients [15,16,17,18,19,20,21]. The first-line treatment for VLS is potent topical corticosteroids, most commonly clobetasol propionate 0.05% ointment applied once daily for 12 weeks. This therapy significantly improves symptoms, reduces inflammation and limits the development of skin lesions. Once remission is achieved, maintenance treatment is recommended. This usually involves less frequent use of corticosteroids, e.g., 1–2 times a week, or an ‘as needed’ regimen. If ineffective or intolerable, topical calcineurin inhibitors may be used [22,23,24]. In cases resistant to traditional treatment with topical glucocorticosteroids, photodynamic therapy (PDT) is an alternative or complementary treatment for VLS. PDT for the treatment of lichen sclerosus is explicitly included in the European Dermatology Forum Guidelines on Topical Photodynamic Therapy 2019 (Part 2), where it is listed among the clinical recommendations [25]. Clinical studies have shown that PDT with 5-aminolevulinate (5-ALA) can improve symptoms such as itching and pain, as well as improve quality of life and improve skin lesions. 5-ALA is a well-established topical photosensitizer used in PDT. After topical application, it is metabolized to protoporphyrin IX, a photoactive compound that accumulates intracellularly in treated lesions and, after illumination with light, generates reactive oxygen species that induce selective cellular damage. In dermatologic PDT, 5-ALA has a favorable, predominantly local safety profile and clinical efficacy comparable to other established topical photosensitizers such as methyl aminolevulinate (MAL) [26]. To perform the procedure, 5-ALA is applied to the lesions, followed by light with a wavelength of 630–635 nm. With a light dose of 80–150 J/cm^2^ per session, typical protocols involve 3–6 sessions at 1–2 week intervals. One session lasts approximately 10–20 min. Transient pain, erythema, and edema are the most common adverse effects [27,28,29].

The successful implementation of PDT requires not only advanced technical infrastructure but also extensive clinical expertise in patient qualification and treatment protocols. Our center, the Center of General and Oncological Dermatology in Wroclaw, has established significant experience in the management of various dermatological conditions using this modality. The clinical practice at our center encompasses a broad range of indications for PDT, demonstrating its versatility in treating both neoplastic and inflammatory skin diseases. The spectrum of conditions routinely managed in our facility includes actinic keratosis (AK), porokeratosis, and superficial basal cell carcinoma (sBCC). Furthermore, our expertise extends to the treatment of chronic inflammatory and viral conditions, such as VLS, erosive lichen planus, and recalcitrant viral warts. This extensive institutional background provides a robust foundation for the current study, ensuring that patient selection and therapeutic procedures are performed according to the highest clinical standards.

## 2. Materials and Methods

### 2.1. Participants

Data collected between June 2025 and February 2026 at the Center of General and Oncological Dermatology in Wroclaw, at the University Center for General and Oncological Dermatology of the Medical University of Wrocław were retrospectively analyzed.

The recruitment process covered a group of 40 patients suffering from VLS and undergoing PDT. Due to the refusal of three individuals to participate in the study, data obtained from 37 patients were analyzed. These were women aged between 25 and 87 (mean 61.08 ± 14.4).

The inclusion criteria for the study were a minimum age of 18 years and a confirmed diagnosis of VLS, established through either clinical presentation or histopathological examination, while exclusion criteria included photodermatoses, pregnancy and breastfeeding, active infections at the treatment site, and the use of photosensitizing medications. Of the 37 patients participating in the study, 17 (46%) were diagnosed on the basis of histopathological examination, and 20 (54%) on the basis of clinical presentation as assessed by dermatologists. Due to the failure of previous treatment methods—topical clobetasol propionate (0.05%) and topical tacrolimus (0.1%)—the patients were qualified for PDT and underwent treatment. During the course of treatment, they were asked to complete questionnaires describing their quality of life (Dermatology Life Quality Index, DLQI) and sexual functioning (Female Sexual Function Index, FSFI). To capture the dynamics of clinical improvement, these assessments were performed at baseline (prior to the first procedure) and 4–6 weeks after each subsequent treatment session. Thirty-seven patients completed the DLQI, while three patients refused to complete the FSFI without giving a reason, and 14 declared no sexual activity. PDT is a method of treating VLS used at the University Center for General and Oncological Dermatology. The study was evaluated by the Bioethics Committee of Wroclaw Medical University as not requiring a resolution of the Bioethics Committee (certificate no. 474/2025).

### 2.2. Treatment Protocol

#### 2.2.1. Pre-Treatment Procedure and Photosensitization

Prior to the procedure, the affected vulvar area was prepared by thorough cleaning and disinfection. A thin layer of a photosensitizing gel (Ameluz^®^, Leverkusen, Germany, containing 5-aminolevulinic acid hydrochloride; 5-ALA HCl, 78 mg/g), corresponding to 10% pure 5-ALA, was applied to the lesions using a sterile spatula [30]. This lecithin-based 10% 5-ALA nanoemulsion has a mean particle diameter of approximately 30 nm, which is associated with high epidermal affinity and improved skin penetration. Following application, the area was protected with an occlusive dressing (aluminum foil) to prevent light degradation and ensure optimal absorption. The incubation period lasted three hours.

#### 2.2.2. Analgesic Management

To manage procedure-related discomfort, systemic analgesia was administered one hour prior to light exposure. Patients received either 1 g of paracetamol or a fixed-dose combination of tramadol (37.5 mg) and paracetamol (325 mg), depending on individual clinical requirements and pain threshold.

#### 2.2.3. Light Irradiation (PDT)

Following the incubation period (3 h), the dressing was removed, and any residual gel was meticulously cleansed. To ensure patient safety and prevent irritation of the urinary tract, the urethral orifice was carefully covered and protected prior to irradiation. Irradiation was performed using the BF-RhodoLED system (Biofrontera AG, Leverkusen, Germany), a medical device specifically registered and optimized for PDT. The affected tissues were exposed to red light with a central wavelength of approximately 635 nm. The treatment followed a standardized clinical protocol, delivering a cumulative light dose (energy density) of 37 J/cm^2^ at an irradiance of a maximum of 77 mW/cm^2^ ±15%. The duration of each session was 10 min. To mitigate thermal sensations and enhance patient tolerance, the device’s integrated cooling fan system was utilized throughout the irradiation process.

#### 2.2.4. Post-Procedural Care and Monitoring

Immediately following treatment, a cooling ointment or a skin-regenerating preparation was applied to the treated area. Patients were instructed to maintain strict intimate hygiene using mild, non-irritating agents. The therapeutic regimen consisted of 1 to 6 sessions per patient, and the total number of sessions was recorded to assess the dose–response relationship.

#### 2.2.5. Safety and Side Effects

The most frequently reported adverse effects included transient intra-procedural pain, as well as post-procedural edema and erythema, all of which typically resolved within several days. Although side effects were monitored for safety purposes, they were not subject to a detailed quantitative recording protocol in this study.

#### 2.2.6. Treatment Duration

The treatment sessions were performed at intervals of 4 to 6 weeks. The number of PDT sessions varied among participants, ranging from 0 to 6, depending on the individual clinical response and the patient’s adherence to the therapy protocol. This variation in the number of procedures allowed for a detailed correlation analysis between treatment intensity and the improvement in quality of life and sexual function.

### 2.3. Instruments

In searching for suitable questionnaires, several patient functional assessment tools were analyzed.

Questionnaires used to assess the quality of life of patients with VLS:

- Dermatology Life Quality Index (DLQI) [16]
- World Health Organization Five-Item Well-Being Index (WHO-5) [16]
- Pictorial Representation of Illness and Self-Measure (PRISM) [31]
- Work Productivity and Activity Impairment: General Health (WPAI:GH) [19]

Questionnaires used to assess the sexual functioning of patients with VLS:

- Female Sexual Function Index (FSFI) [19]
- Female Sexual Distress Scale (FSDS) [17]
- Sexual Quality of Life-Female (SQOL-F) [19]
- Female Genital Self-Image Scale (FGSIS) [18].

We selected the DLQI and FSFI questionnaires for our research. Both have been validated among dermatology patients in Poland and are available in Polish. The DLQI scale is used in everyday clinical practice and in scientific research. The FSFI scale is recommended for assessing the sexual functioning of patients with lichen sclerosus [12,15,16,17,18,19].

The DLQI questionnaire consists of ten questions concerning the impact of skin disease on various aspects of the patient’s life over the past seven days. Symptoms, feelings, daily functioning, social relationships, occupational activity and sexual life are all elements that are examined. The score for each question ranges from 0 (not at all) to 3 (very much), and the total score from 0 to 30 reflects the degree of deterioration in quality of life. A score of 0–1 indicates no impact on quality of life, a score of 2–5 indicates a small impact, a score of 6–10 indicates a moderate impact, a score of 11–20 indicates a large impact, and a score of 21–30 indicates a very large impact on quality of life [32].

In 2004, Prof. Adam Reich, MD, PhD, and his team from the Department and Clinic of Dermatology at the Medical University of Rzeszów developed a Polish version of the DLQI scale in accordance with international standards for the translation and validation of quality of life assessment tools [33,34].

The FSFI questionnaire consists of 19 questions and assesses women’s sexual functioning in six categories: desire, arousal, lubrication, orgasm, satisfaction and pain during intercourse. Each domain is assessed using a set of questions, and answers are rated on a scale of 0 to 5 points. Higher scores on the scale indicate better sexual functioning. The scores from each domain are used to calculate the total score; scores below a certain threshold (below 27.5 in the Polish population) indicate the presence of sexual dysfunction. Dr Krzysztof Nowosielski and his research team developed the Polish version of the FSFI scale in 2013 [35].

### 2.4. Data Analysis

Statistical procedures were executed in two distinct phases, considering the specific characteristics of the research tools and the study population. The assessment of the impact of dermatological conditions on general quality of life (DLQI) was conducted on the total cohort of enrolled patients (N = 37). Conversely, the analysis of sexual function, based on the FSFI questionnaire, was restricted to a subset of 20 patients who reported being sexually active during the four weeks preceding the study. Excluding sexually inactive individuals was essential to prevent systematic bias in FSFI scoring, where a lack of activity is recorded as “0,” potentially leading to a misinterpretation of actual clinical dysfunction.

Given the ordinal nature of the Likert-type scales and the group sizes, the non-parametric Kendall’s tau (τ) rank correlation coefficient was employed to evaluate relationships between pairs of variables. This method was selected for its robustness against outliers and higher precision in small sample sizes compared to Spearman’s correlation. The direction of the associations was determined by the sign of the τ coefficient (positive for direct correlations, negative for inverse correlations).

The initial level of statistical significance was set at *p* = 0.05. To address the multiple testing burden and mitigate the risk of Type I error inflation, the False Discovery Rate (FDR) correction was applied to the resulting *p*-values using the Benjamini–Hochberg procedure. This approach ensured rigorous control over the proportion of false-positive findings. The results are presented in a summary correlation table, with key associations further visualized through distribution and scatter plots. All calculations were performed using R version 4.5.2.

## 3. Results

The statistical analysis was conducted in two phases based on the relevance of the clinical tools. Quality of life assessment (DLQI) was performed in the entire cohort of 37 patients. Analysis of sexual function, including the total FSFI score and its individual domains, was restricted to a subset of 20 patients who reported being sexually active. The results are presented in a summary format (correlation tables) and a detailed format, including distribution and scatter plots to visualize relationships among variables (Table 1).

**Figure 1:**
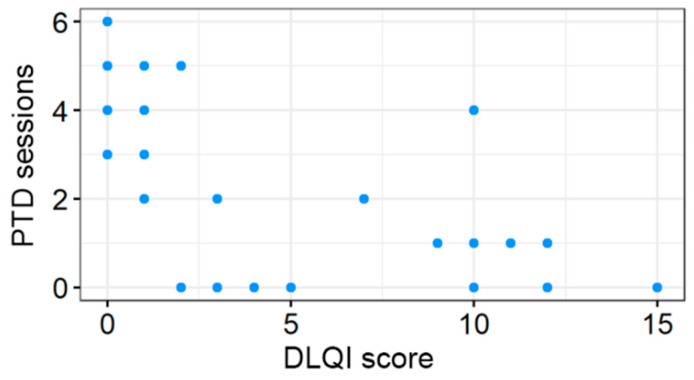
Correlation between the number of PDT sessions and DLQI score.

Statistical analysis revealed a strong and significant correlation between the number of PDT sessions and clinical outcomes. A robust negative correlation was observed between the number of PDT procedures and the DLQI score (τ = −0.583; adjusted *p* < 0.001), as shown in Figure 1, indicating that a higher number of treatments is significantly associated with improved overall quality of life.

**Figure 2:**
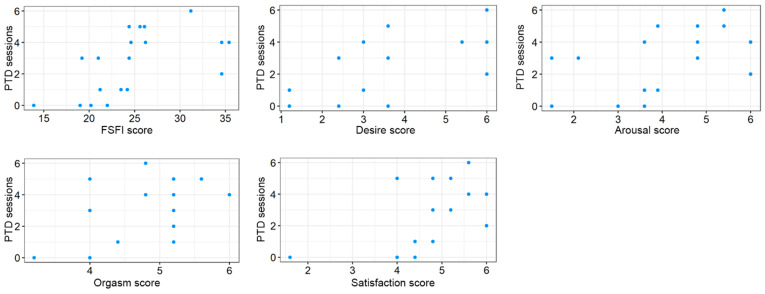
Correlations between the number of PDT sessions and the total FSFI score, and its domains.

Regarding sexual health, a significant positive correlation was identified between the cumulative number of PDT sessions and the total FSFI score (τ = 0.556; adjusted *p* = 0.014). Detailed analysis of FSFI domains, as depicted in Figure 2, showed that PDT sessions were significantly associated with improvements in:

- Desire: τ = 0.512; adjusted *p* = 0.016
- Arousal: τ = 0.473; adjusted *p* = 0.020
- Orgasm: τ = 0.487; adjusted *p* = 0.020
- Satisfaction: τ = 0.507; adjusted *p* = 0.016

**Figure 3:**
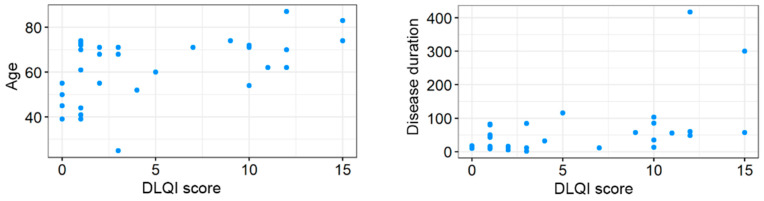
Correlations between age and disease duration versus DLQI score.

Age was identified as a critical factor modulating both quality of life and sexual function. A positive correlation was observed between age and DLQI scores (τ = 0.360; adjusted *p* = 0.016), suggesting that older patients experience a greater disease burden. Conversely, age was negatively correlated with the total FSFI score (τ = −0.392; adjusted *p* = 0.040), with the strongest negative impact seen in the domains of Desire (τ = −0.504; adjusted *p* = 0.016) and Satisfaction (τ = −0.504; adjusted *p* = 0.016). Disease duration was significantly correlated with general quality of life (DLQI: τ = 0.321; adjusted *p* = 0.020), indicating that patients with a longer history of symptoms report lower quality of life. However, disease duration did not show a statistically significant relationship with total FSFI scores or individual sexual function domains (adjusted *p* > 0.05). Correlations between age and disease duration versus DLQI score are shown in Figure 3.

## 4. Discussion

VLS is a long-term inflammatory skin condition that primarily affects postmenopausal women; however it can also afflict prepubescent girls and women of any age. The vulvar and perianal areas are particularly affected. Although the exact cause is unknown, hypoestrogenic conditions, hereditary susceptibility, and autoimmune processes are all suggested. Clinically, it manifests as white, thin, atrophic plaques that frequently involve the vulva and perianal region in a “figure-eight” pattern. Symptoms include intense burning, pruritus, pain, dyspareunia, and, in more severe cases, architectural distortion (regression of labia minora, clitoral phimosis, introital stenosis). Although the diagnosis is mostly made clinically, vulvar biopsy is advised to confirm the diagnosis or rule out cancer, particularly in light of the elevated risk of vulvar squamous cell carcinoma (estimated lifetime risk 2–6%). High-potency topical corticosteroid ointment, such as clobetasol propionate 0.05%, is the first-line therapy because it effectively controls symptoms, prevents scarring, and lowers the risk of malignancy. The chronicity, recurrence risk, and possibility of malignant change necessitate long-term monitoring [6,36].

VLS is treated using PDT, especially when conventional therapy is inadequate or inappropriate or when the patient is resistant to glucocorticosteroid treatment. 5-ALA topically as a photosensitizer and then exposing the area to red light with a wavelength of 630–635 nm is the most popular method. According to studies, PDT can significantly enhance quality of life and clinical symptoms (pain, itching, skin lesions), as well as reduce lymphocytic infiltration and improve histological markers. Erythema, swelling, and temporary discomfort at the treatment site are typically the only side effects [27,28,29,37]. Evidence on the effect of PDT on quality of life and sexual function in VLS remains limited, as the available literature is largely based on small single-center studies with heterogeneous treatment protocols and outcome measures. Recent reviews note that most published PDT series include only 10–30 patients, although the overall evidence base is growing [13]. In our study, which included 37 women with refractory VLS, a greater number of PDT sessions was associated with lower DLQI scores and better overall FSFI results, particularly in the domains of desire, arousal, orgasm, and satisfaction. Compared with earlier reports, our cohort was larger than the 10-patient series by Lan et al. and the 30-patient studies by Zhang et al., all of which also demonstrated improvements in quality of life and sexual outcomes after ALA-PDT [27,38,39]. Our findings are also consistent with the more recent prospective study by Chen et al., which included 60 patients and showed sustained improvement in DLQI after six sessions [40]. Together, these data support PDT as a promising option for refractory VLS, while underscoring the need for larger, standardized prospective studies.

The application of a photosensitizer (usually 5-ALA) topically to the vulvar skin’s affected regions is the first step in PDT VLS. The affected areas are then exposed to red light with a wavelength of 630–635 nm. Following the application of 5-ALA (often at a 20% concentration), the skin is blocked for three to four hours to allow the medication to enter the body. After that, the subject is exposed to radiation for 15–20 min at an intensity of 100–150 J/cm^2^. Every two weeks, the process is repeated, often in a sequence of three to six treatments. The production of reactive oxygen species is the basis for the mechanism of action, which modifies the local immune response and selectively destroys diseased skin cells. Quality of life is increased, clinical symptoms including pain and itching are lessened, microcirculation is enhanced, and lymphocytic infiltration is decreased [27,28,29,41].

Our study involved 37 adult patients (mean age 61.08; pm 14.4) with VLS who had previously failed standard topical therapies (0.05% clobetasol propionate and 0.1% tacrolimus). The treatment utilized a standardized, high-efficiency PDT protocol at the Center of General and Oncological Dermatology in Wrocław, employing Ameluz^®^ (10% 5-ALA) in conjunction with the BF-RhodoLED system (635 nm, 37 J/cm^2^, approx. 77 mW/cm^2^). This protocol was specifically designed to maximize therapeutic impact through a condensed, 10 min irradiation period. The therapeutic regimen was designed to assess a dose–response relationship, with participants undergoing 1 to 6 sessions at 4–6 week intervals. Inclusion criteria required a minimum age of 18 years and a confirmed VLS diagnosis, while exclusion criteria consisted of photodermatoses, pregnancy and breastfeeding, active infections at the treatment site, and the use of photosensitizing medications. To ensure clinical precision, the urethral orifice was protected during irradiation, and systemic analgesia was provided to enhance tolerance. Quality of life and sexual health were evaluated using validated Polish versions of the DLQI (N = 37) and FSFI (n = 20) questionnaires. Notably, 14 patients declared sexual inactivity and three refused the FSFI, emphasizing the significant psychosexual burden of VLS. Statistical integrity was maintained by using Kendall’s τ—a robust measure for ordinal data and smaller samples—and applying the Benjamini–Hochberg (FDR) correction to control for Type I errors across multiple domain analyses.

The results of our study provide compelling evidence that PDT, delivered via these optimized parameters, is highly effective in improving both the general quality of life and the sexual health of patients suffering from VLS.

### 4.1. Therapeutic Impact on Quality of Life: Efficiency of the High-Precision Protocol

The strong correlation between the number of PDT procedures and the reduction in DLQI scores (τ = −0.583, adjusted *p* < 0.001) serves as a primary indicator of the protocol’s efficacy. Our data suggest that the 77 mW/cm^2^ irradiance effectively modulates the vulvar microenvironment, with repeated exposure being the key driver of success. Furthermore, the correlation between disease duration and DLQI (τ = 0.321, adjusted *p* = 0.020) highlights a critical “window of opportunity”. The efficacy of our 10 min irradiation protocol in achieving significant results, even in chronic cases, suggests that it may represent a promising therapeutic option that warrants further evaluation earlier in the disease course, before irreversible scarring occurs. Interestingly, the lack of correlation between disease duration and FSFI scores (τ = −0.026) suggests that while sexual dysfunction may plateau early, the quality of life continues to deteriorate, reinforcing the need for the prompt initiation of our optimized PDT regimen.

### 4.2. Restoration of Sexual Function and the “Desire” Paradox

A noteworthy finding is the efficacy of our protocol in restoring physiological sexual responses. Significant correlations were found between the number of PDT sessions and domains such as Arousal (τ = 0.473), Orgasm (τ = 0.487), and Satisfaction (τ = 0.507). These results demonstrate that the high-precision LED delivery system does more than alleviate irritation; it likely improves tissue elasticity and restores genital sensitivity. The significant positive correlation with Desire (τ = 0.512, adjusted *p* = 0.016) is particularly telling. It suggests that our specific PDT parameters effectively break the “pain-avoidance” cycle, allowing for a rebound in libido as the chronic physical burden is lifted.

### 4.3. Biological Barriers and the Influence of Age

Despite the high efficacy of the protocol, age emerged as an independent modulator. A positive correlation between age and DLQI (τ = 0.360, adjusted *p* = 0.016) and a negative correlation with sexual well-being (τ = −0.504) reflect the “overlap” of VLS with genitourinary syndrome of menopause (GSM). The non-significant findings in the Lubrication and Pain domains further validate the specificity of our protocol; while PDT effectively treats the vulvar integument, it does not replace the hormonal components of lubrication. The stability of pain scores suggests that in older patients, structural changes may require a multi-modal approach, yet the high-precision PDT remains the cornerstone of surface tissue restoration.

### 4.4. Methodological Rigor and Protocol Validation

The robustness of our conclusions is cemented by the application of the Benjamini–Hochberg (FDR) correction. The fact that the correlations between our 10 min, 77 mW/cm^2^ PDT sessions and key sexual function domains remained statistically significant after adjusting for multiple comparisons confirms that the observed improvements are a direct result of the therapy. This statistical integrity validates our protocol as a reliable and effective standard for treating refractory VLS.

When situating our results within the broader clinical landscape, it is evident that while the choice of a 635 nm ± 15 nm red light source is consistent across most studies focusing on VLS, there is significant heterogeneity regarding the total light dose and irradiance [27,28,38,42,43,44]. Many researchers employ the same wavelength to target protoporphyrin IX (PpIX) accumulation; however, our protocol’s use of a cumulative energy density of 37 J/cm^2^ at an irradiance of maximum 77 mW/cm^2^ ±15% differs from several other reported regimens. Our findings stand in notable contrast to protocols frequently cited in the literature. For example, Cao et al. utilized a significantly more intensive regimen involving a 20% 5-ALA formulation—double the concentration used in our study—with a power density of 60–80 mW/cm^2^ and an exposure time of 30 min per session [42]. Similarly, Qu et al. employed a high-intensity approach, using cotton sheets soaked in 20% 5-ALA for a 3-to-4 h incubation period, followed by 30–40 min of irradiation at 60–80 mW/cm^2^. While they reported improvements using the Cattaneo clinical symptom and sign score, their treatment frequency was significantly higher, with intervals of only 10 days between the 3–6 sessions [44]. Furthermore, Kohn et al. employed 20% 5-ALA with irradiation at 60–90 mW/cm^2^ for 20 min, delivering 100–150 J/cm^2^ per session [23]. Li et al. implemented an even more aggressive protocol with 20% 5-ALA, 80 J/cm^2^, 80 mW/cm^2^, and 30 min irradiation times, reporting that the therapy was “mildly toxic” for most patients [43]. Lan et al., while using a 10% 5-ALA concentration similar to ours, employed a power density of 100 mW/cm^2^ for 20 min—substantially more aggressive irradiation parameters [38]. A particularly striking comparison is found in the work of Imbernón-Moya et al. [45], who utilized MAL at a dose of 37 J/cm^2^. While their total energy dose was identical to ours, they applied a much higher intensity of 70 mW/cm^2^. This necessitated sedation with midazolam for most patients due to moderate-to-severe pain, and in two cases, general anesthesia with propofol was required due to excruciating pain [38]. In contrast, our study achieved comparable therapeutic goals using the BF-RhodoLED system (max. approx. 77 mW/cm^2^ ±15% for 10 min), which was well-tolerated without the need for systemic sedation or anesthesia. This suggests that the thermal sensation and subsequent pain are highly dependent on the irradiance (mW/cm^2^) rather than just the total energy dose. Despite their use of higher concentrations and nearly triple our irradiation duration, our study demonstrated that comparable clinical success in improving patient quality of life and sexual function can be achieved with a more refined approach. Specifically, our use of the BF-RhodoLED system (40 mW/cm^2^ for 10 min) and 10% 5-ALA (Ameluz^®^) proved highly effective, suggesting that the nanoemulsion delivery system may compensate for lower active ingredient concentrations. This optimized delivery allows for a significant reduction in physical light parameters and treatment frequency without compromising the cumulative therapeutic effect.

### 4.5. Technical Advantages of Our Protocol

#### Optimized Irradiance and Improved Tolerability

The choice of approximately 77 mW/cm^2^ in our center is noteworthy as it balances therapeutic efficacy with efficient treatment times. Crucially, these specific parameters, utilized in combination with the BF-RhodoLED system and 10% 5-ALA nanoemulsion, are strictly aligned with current European guidelines for the treatment of AK [46,47]. By adopting this established dermatological standard for the treatment of VLS, we ensured that our patients received a regimen with a well-characterized safety profile and optimized light-to-drug interaction. While this irradiance is higher than in some experimental settings, our patients’ ability to complete up to six sessions without systemic anesthesia suggests that the 10 min duration and the spectral stability of the BF-RhodoLED system provide a favorable safety profile. High irradiance (e.g., 100 mW/cm^2^ as used by Lan et al.) combined with longer exposure times is often associated with a higher risk of pain and thermal damage to the delicate vulvar mucosa [38]. Our refined approach demonstrates that clinical success in improving patient quality of life and sexual function can be achieved by optimizing the delivery system and limiting the duration of light exposure.

### 4.6. Nanoemulsion Formulation

A significant point of differentiation between our methodology and many previous clinical trials lies in the concentration of the photosensitizer. Historically, the majority of studies investigating PDT for VLS have utilized a 20% concentration of 5-ALA, often prepared as a magistral formulation [27,28]. The clinical efficacy observed in our results is particularly compelling given this lower concentration. This can be attributed to the nanoemulsion formulation of Ameluz^®^, which is specifically designed to enhance the penetration of 5-ALA into the deeper layers of the epidermis and dermis more effectively than traditional 20% 5-ALA creams. This advanced delivery system ensures that a lower concentration of the active ingredient can still achieve a high level of protoporphyrin IX (PpIX) accumulation [48]. Furthermore, our approach differs from researchers like Imbernón-Moya et al., who utilized MAL at a concentration of 160 mg/g. While MAL is a lipophilic derivative of 5-ALA designed to improve skin penetration, it often requires long incubation periods (3 h) and, as seen in their study, was associated with significant pain at an irradiance of 70 mW/cm^2^, requiring sedation or even general anesthesia [45]. Notably, our protocol utilized a slightly higher irradiance (approx. 77 mW/cm^2^), yet it was well-tolerated without any systemic sedation. This suggests that the 10% 5-ALA nanoemulsion, when activated by a precise, narrow-band LED source (BF-RhodoLED), provides an optimal therapeutic window. It achieves sufficient PpIX accumulation to induce significant clinical effects while avoiding the extreme phototoxicity and thermal discomfort reported with other formulations and light sources. This underscores the importance of the delivery vehicle (nanoemulsion) and the spectral purity of the light source over the absolute concentration or the specific chemical derivative (5-ALA vs. MAL). By achieving comparable or superior clinical outcomes with a vastly improved safety profile, our protocol is better suited for the repeated sessions required for chronic conditions like VLS.

### 4.7. Shortened Irradiation Time

Another critical technical discrepancy observed in the literature pertains to the duration of the irradiation phase. In many clinical trials, the duration of a single PDT session was significantly longer than in our study—often reaching 20–30 min to deliver the required energy dose [27,42]. In contrast, our protocol utilized the BF-RhodoLED system, which allowed for the delivery of a cumulative light dose of 37 J/cm^2^ in exactly 10 min. This efficiency is a direct result of the optimized irradiance (approx. 77 mW/cm^2^) provided by the LED array, which maintains a constant and uniform output. The ability to deliver the therapeutic dose in a shorter time frame compared to many conventional protocols is a substantial clinical advantage. It enhances the overall patient experience by minimizing the time spent in a potentially uncomfortable position and significantly improves the throughput and efficiency of the clinical setting. Most importantly, as our results demonstrate, this higher intensity—when delivered via a precise LED source—does not compromise patient tolerability, allowing for a swift yet effective treatment session.

### 4.8. Extended Treatment Intervals

A notable distinction in our therapeutic protocol concerns the temporal distribution of the sessions. A review of current literature reveals that many researchers perform PDT treatments with high frequency, often at intervals of 1 to 2 weeks, typically completing a series of 3 to 6 sessions in a very short timeframe [28,39]. In contrast, our study employed a more spaced-out approach, with procedures performed every 4 to 6 weeks. Despite these longer intervals, our results demonstrated a significant, cumulative improvement in both quality of life and sexual function, correlated with the total number of sessions (ranging from 1 to 6). This suggests several clinical advantages: the 4-to-6-week interval aligns more closely with the natural cycle of epidermal regeneration and inflammatory resolution, potentially minimizing the risk of cumulative local irritation in the sensitive vulvar region. Given that VLS predominantly affects older populations (mean age of 61.08 in our study), longer intervals between visits significantly reduce the logistical burden on the patient, making the long-term, multi-session therapy more sustainable and less disruptive to their daily lives.

### 4.9. Clinical Implications

The fact that our study demonstrated a significant improvement in DLQI and FSFI using these specific parameters suggests that a 37 J/cm^2^ dose delivered at a high, yet precise irradiance (~77 mW/cm^2^) is highly effective for vulvar tissue. This adds a valuable data point to the ongoing discussion regarding the standardization of PDT protocols, suggesting that clinical success can be achieved through optimized, shorter irradiation periods (10 min) without the need for systemic sedation or anesthesia. Our results demonstrate that a more moderate approach using Ameluz^®^ (10% 5-ALA) and a 10 min irradiation period (37 J/cm^2^) can achieve a similarly profound impact on quality of life as high-dose protocols. By delivering nearly half the energy dose per session compared to more aggressive regimens, we minimize the “toxicity” and thermal discomfort often associated with high-power density, thereby potentially improving patient adherence to the long-term therapeutic process. Our findings advocate for a “marathon rather than a sprint” approach, where the total number of procedures (the cumulative dose) is the primary driver of recovery, even when the sessions are distributed over a longer chronological period. By achieving significant therapeutic milestones with sessions spaced further apart, we demonstrate that high-frequency protocols—which may be difficult for many clinics and patients to maintain—are not strictly necessary to achieve profound improvements in DLQI and FSFI scores. The identification of age as an independent modulator of treatment response has important clinical implications. Older patients may benefit from adjunctive hormonal therapy to address the concurrent GSM component, while the “window of opportunity” concept emphasizes the importance of early, aggressive intervention before irreversible structural changes occur.

### 4.10. Limitations

Several limitations of this study should be noted. The relatively small sample size limits the generalizability of the findings. In addition, procedural photographs and standardized before/after clinical images were not systematically collected, precluding visual documentation of treatment outcomes. Moreover, because VLS lacks a universally accepted formal staging system and no formal severity scoring tool was used in this study, treatment efficacy could not be stratified by disease severity. Notably, all included patients had persistent disease despite previous treatment, indicating a difficult-to-treat population. Future studies should address these limitations by including larger cohorts, photographic documentation, and structured severity assessment.

## 5. Conclusions

Our study provides strong evidence that PDT, when delivered via a refined and patient-centered protocol, is a highly effective intervention for patients with VLS who are refractory to standard topical corticosteroid and calcineurin inhibitor therapies. By utilizing a 10% 5-ALA nanoemulsion (Ameluz^®^) in conjunction with an optimized, high-precision LED light regimen (37 J/cm^2^ at approx. 77 mW/cm^2^), we have demonstrated that clinical success can be achieved without the treatment-limiting pain or local toxicity often reported in protocols with longer exposure times or higher-concentration formulations. This approach offers a favorable balance between therapeutic efficacy, patient comfort, and practical feasibility, emphasizing that high-precision light delivery and shortened irradiation times (10 min)—rather than excessively aggressive regimens—can ensure excellent patient adherence. Furthermore, the cumulative number of sessions emerges as the primary driver of clinical improvement. Future studies should focus on long-term follow-up, optimal maintenance regimens, and the potential synergy between PDT and hormonal therapies in postmenopausal patients.

## Figures and Tables

**Table 1:** Correlation table between studied variables, such as DLQI score, FSFI total score and FSFI domains, disease duration, and number of PDT sessions.

Variables	τ	*p*-Value	Data	Adjusted *p*-Value *
DLQI and PDT	−0.5831	<0.0001	all data	0.0002
FSFI and PDT	0.5562	0.0012	with FSFI	0.0143
DLQI and age	0.36054	0.0035	all data	0.0166
Desire and PDT	0.5125	0.0048	with FSFI	0.0166
Desire and age	−0.504	0.0043	with FSFI	0.0166
Satisfaction and PDT	0.5075	0.0044	with FSFI	0.0166
Satisfaction and age	−0.5045	0.0036	with FSFI	0.0166
Arousal and PDT	0.4734	0.0075	with FSFI	0.0201
Orgasm and PDT	0.4878	0.0068	with FSFI	0.0201
DLQI and disease duration	0.32124	0.0087	all data	0.0201
FSFI and age	−0.3924	0.0185	with FSFI	0.0404
Lubrication and disease duration	0.3063	0.0771	with FSFI	0.1542
Arousal and age	−0.2898	0.0919	with FSFI	0.1697
Pain and disease duration	0.2691	0.1378	with FSFI	0.23621
Satisfaction and disease duration	−0.211	0.1984	with FSFI	0.3174
Lubrication and PDT	0.2019	0.264	with FSFI	0.3960
Desire and disease duration	−0.175	0.3155	with FSFI	0.4454
Orgasm and age	−0.164	0.3489	with FSFI	0.4652
Lubrication and age	−0.1234	0.4818	with FSFI	0.6086
Pain and age	−0.0977	0.5946	with FSFI	0.7135
Orgasm and disease duration	−0.0749	0.665	with FSFI	0.751
FSFI and disease duration	−0.0268	0.8707	with FSFI	0.8707
Arousal and disease duration	0.0335	0.8435	with FSFI	0.8707
Pain and PDT	0.0407	0.8298	with FSFI	0.8707

DLQI, Dermatology Life Quality Index; FSFI, Female Sexual Function Index; PDT, photodynamic therapy. * Adjusted for age and the number of comparisons.

## Data Availability

The data supporting the findings of this study are not publicly available due to ethical, legal, and privacy restrictions.

## Abbreviations

The following abbreviations are used in this manuscript:

5-ALA	5-aminolevulinate
AK	Actinic keratosis
DLQI	Dermatology Life Quality Index
FDR	False discovery rate
FGSIS	Female Genital Self-Image Scale
FSDS	Female Sexual Distress Scale
FSFI	Female Sexual Function Index
GSM	Genitourinary Syndrome of Menopause
MAL	Methyl aminolevulinate
PDT	Photodynamic therapy
PRISM	Pictorial Representation of Illness and Self-Measure
sBCC	Superficial basal cell carcinoma
SQOL-F	Sexual Quality of Life-Female
VLS	Vulvar lichen sclerosus
WHO-5	World Health Organization Five-Item Well-Being Index
WPAI:GH	Work Productivity and Activity Impairment: General Health

## References

[1] Xie X., Wu K. (2024). Advances in the pathogenesis of vulvar lichen sclerosus. Mol. Biol. Rep..

[2] Lee A., Fischer G. (2018). Diagnosis and Treatment of Vulvar Lichen Sclerosus: An Update for Dermatologists. Am. J. Clin. Dermatol..

[3] Perez-Lopez F.R., Vieira-Baptista P. (2017). Lichen sclerosus in women: A review. Climacteric.

[4] Guidozzi F. (2021). Lichen sclerosus of the vulva. Climacteric.

[5] Orszulak D., Dulska A., Nizinski K., Skowronek K., Bodziony J., Stojko R., Drosdzol-Cop A. (2021). Pediatric Vulvar Lichen Sclerosus-A Review of the Literature. Int. J. Environ. Res. Public Health.

[6] Liu L., He Y., Hu Q., Sun K., Yang M., Chang J. (2024). Vulvar lichen sclerosus in girls and adult females: A single-center retrospective study of 744 patients in China. J. Dermatol..

[7] De Luca D.A., Papara C., Vorobyev A., Staiger H., Bieber K., Thaçi D., Ludwig R.J. (2023). Lichen sclerosus: The 2023 update. Front. Med..

[8] Terlou A., Santegoets L.A., van der Meijden W.I., Heijmans-Antonissen C., Swagemakers S.M., van der Spek P.J., Ewing P.C., van Beurden M., Helmerhorst T.J., Blok L.J. (2012). An autoimmune phenotype in vulvar lichen sclerosus and lichen planus: A Th1 response and high levels of microRNA-155. J. Investig. Dermatol..

[9] Wang L., Lv Q., Guo J., Wang J., Pan J. (2022). Transcriptome Profiling and Network Analysis Provide Insights Into the Pathogenesis of Vulvar Lichen Sclerosus. Front. Genet..

[10] Tran D.A., Tan X., Macri C.J., Goldstein A.T., Fu S.W. (2019). Lichen Sclerosus: An autoimmunopathogenic and genomic enigma with emerging genetic and immune targets. Int. J. Biol. Sci..

[11] Paganelli A., Didona D., Scala E. (2025). Cytokine Networks in Lichen Sclerosus: A Roadmap for Diagnosis and Treatment?. Int. J. Mol. Sci..

[12] Sun P., Kraus C.N., Zhao W., Xu J., Suh S., Nguyen Q., Jia Y., Nair A., Oakes M., Tinoco R. (2026). Spatial and Single-Cell Transcriptomics Reveal Keratinocytes as Key Players in Vulvar Lichen Sclerosus Pathogenesis. J. Investig. Dermatol..

[13] Beutler K., Jankowska-Konsur A., Nowicka D. (2026). Photodynamic Therapy in the Treatment of Vulvar Lichen Sclerosus: Systematic Review. Dermatol. Ther..

[14] Liu T., Zhang Y., Sun K., Shao Y., Yang K., Yang M., Chang J. (2026). Clinical spectrum and quality of life burden of vulvar lichenoid dermatoses: A cross-sectional study of 1,177 patients. Clin. Exp. Dermatol..

[15] Pope R., Lee M.H., Myers A., Song J., Abou Ghayda R., Kim J.Y., Hong S.H., Lee S.B., Koyanagi A., Jacob L. (2022). Lichen Sclerosus and Sexual Dysfunction: A Systematic Review and Meta-Analysis. J. Sex Med..

[16] Vittrup G., Morup L., Heilesen T., Jensen D., Westmark S., Melgaard D. (2022). Quality of life and sexuality in women with lichen sclerosus: A cross-sectional study. Clin. Exp. Dermatol..

[17] Caspersen I.S., Hojgaard A., Laursen B.S. (2023). The influence of lichen sclerosus on women’s sexual health from a biopsychosocial perspective: A mixed methods study. J. Sex Med..

[18] Yildiz S., Cengiz H., Kaya C., Alay I., Ozturk E., Tunca A.F., Erdogan A., Yasar L. (2022). Evaluation of genital self-image and sexual dysfunction in women with vulvar lichen planus or lichen sclerosus. J. Psychosom. Obstet. Gynaecol..

[19] Jablonowska O., Wozniacka A., Szkarlat S., Zebrowska A. (2023). Female genital lichen sclerosus is connected with a higher depression rate, decreased sexual quality of life and diminished work productivity. PLoS ONE.

[20] He Y., Liu L., Yang K., Sun K., Zhang Q., Yang M., Chang J. (2024). Quality of life and burden of disease of vulvar lichen sclerosus: A single-center retrospective study in China. Int. J. Gynaecol. Obstet..

[21] Haefner H.K., Aldrich N.Z., Dalton V.K., Gagne H.M., Marcus S.B., Patel D.A., Berger M.B. (2014). The impact of vulvar lichen sclerosus on sexual dysfunction. J. Womens Health.

[22] Chi C.C., Kirtschig G., Baldo M., Brackenbury F., Lewis F., Wojnarowska F. (2011). Topical interventions for genital lichen sclerosus. Cochrane Database Syst. Rev..

[23] Kohn J.R., Connors T.M., Chan W., Liang C.S., Dao H., Vyas A. (2020). Clinical outcomes and adherence to topical corticosteroid therapy in women with vulvar lichen sclerosus: A retrospective cohort study. J. Am. Acad. Dermatol..

[24] Pergialiotis V., Bellos I., Biliou E.C., Varnava P., Mitsopoulou D., Doumouchtsis S.K. (2020). An arm-based network meta-analysis on treatments for vulvar lichen sclerosus and a call for development of core outcome sets. Am. J. Obstet. Gynecol..

[25] Morton C.A., Szeimies R.M., Basset-Seguin N., Calzavara-Pinton P.G., Gilaberte Y., Haedersdal M., Hofbauer G.F.L., Hunger R.E., Karrer S., Piaserico S. (2020). European Dermatology Forum guidelines on topical photodynamic therapy 2019 Part 2: Emerging indications—Field cancerization, photorejuvenation and inflammatory/infective dermatoses. J. Eur. Acad. Dermatol. Venereol..

[26] Lee Y., Baron E.D. (2011). Photodynamic therapy: Current evidence and applications in dermatology. Semin. Cutan. Med. Surg..

[27] Zhang F., Li D., Shi L., Gu Y., Xu Y., Wu C. (2021). Efficacy of 5-Aminolevulinic Acid (ALA)-Photodynamic Therapy (PDT) in Refractory Vulvar Lichen Sclerosus: Preliminary Results. Med. Sci. Monit..

[28] Shi L., Miao F., Zhang L.L., Zhang G.L., Wang P.R., Ji J., Wang X.J., Huang Z., Wang H.W., Wang X.L. (2016). Comparison of 5-Aminolevulinic Acid Photodynamic Therapy and Clobetasol Propionate in Treatment of Vulvar Lichen Sclerosus. Acta Derm. Venereol..

[29] Gerkowicz A., Szczepanik-Kulak P., Krasowska D. (2021). Photodynamic Therapy in the Treatment of Vulvar Lichen Sclerosus: A Systematic Review of the Literature. J. Clin. Med..

[30] Ameluz Summary of Product Characteristics. https://www.ema.europa.eu/en/medicines/human/EPAR/ameluz.

[31] Borghi A., Odorici G., Scuderi V., Valpiani G., Morotti C., Corazza M. (2020). Measuring perceived benefit and disease-related burden in patients affected with vulvar lichen sclerosus after a standard topical corticosteroid treatment. Results from a cohort study using Pictorial Representation of Illness and Self-measure and Dermatology Life Quality Index. Dermatol. Ther..

[32] Hongbo Y., Thomas C.L., Harrison M.A., Salek M.S., Finlay A.Y. (2005). Translating the science of quality of life into practice: What do dermatology life quality index scores mean?. J. Invest. Dermatol..

[33] Basra M.K., Fenech R., Gatt R.M., Salek M.S., Finlay A.Y. (2008). The Dermatology Life Quality Index 1994–2007: A comprehensive review of validation data and clinical results. Br. J. Dermatol..

[34] Vyas J., Johns J.R., Ali F.M., Ingram J.R., Salek S., Finlay A.Y. (2024). A Systematic Review of 207 Studies Describing Validation Aspects of the Dermatology Life Quality Index. Acta Derm. Venereol..

[35] Nowosielski K., Wrobel B., Sioma-Markowska U., Poreba R. (2013). Development and validation of the Polish version of the Female Sexual Function Index in the Polish population of females. J. Sex Med..

[36] Ringel N.E., Iglesia C. (2020). Common Benign Chronic Vulvar Disorders. Am. Fam. Physician.

[37] Bizon M., Maslinska D., Sawicki W. (2022). Influence of Photodynamic Therapy on Lichen Sclerosus with Neoplastic Background. J. Clin. Med..

[38] Lan T., Zou Y., Hamblin M.R., Yin R. (2018). 5-Aminolevulinic acid photodynamic therapy in refractory vulvar lichen sclerosus et atrophicus: Series of ten cases. Photodiagnosis Photodyn. Ther..

[39] Zhang F., Li D., Shi L., Gu Y., Xu Y. (2020). 5-ALA-photodynamic therapy in refractory vulvar lichen sclerosus et atrophicus. Int. J. Clin. Exp. Pathol..

[40] Chen G., Li Y., Mu J., Lu Z., Cao L., Cheng X., Wang Y. (2025). Analysis of subjective efficacy of 5-aminolevulinic acid photodynamic therapy in the treatment of refractory vulvar lichen sclerosus. Photodiagnosis Photodyn. Ther..

[41] Olejek A., Kozak-Darmas I., Kellas-Sleczka S., Steplewska K., Biniszkiewicz T., Birkner B., Jarek A., Nowak L., Stencel-Gabriel K., Sieron A. (2009). Effectiveness of photodynamic therapy in the treatment of lichen sclerosus: Cell changes in immunohistochemistry. Neuro Endocrinol. Lett..

[42] Cao Y., Qu Z., Sun X., Cui G., Wei H., Wang Z., Lin X. (2024). Evaluation of the therapeutic effects of photodynamic therapy in vulvar lichen sclerosus and impact on patient quality of life and sexual funtion. Photodiagnosis Photodyn. Ther..

[43] Li Z., Wang Y., Wang J., Li S., Xiao Z., Feng Y., Gu J., Li J., Peng X., Li C. (2020). Evaluation of the efficacy of 5-aminolevulinic acid photodynamic therapy for the treatment of vulvar lichen sclerosus. Photodiagnosis Photodyn. Ther..

[44] Qu Z., Lin X., Liu M., Wang J., Wang F., Zhang B., Shen L., Wang Z. (2024). Clinical efficacy analysis of 5-aminolevulinic acid photodynamic therapy for vulvar lichen sclerosus. Photodiagnosis Photodyn. Ther..

[45] Imbernon-Moya A., Martinez-Perez M., Churruca-Grijelmo M., Lobato-Berezo A., Vargas-Laguna E., Fernandez-Cogolludo E., Aguilar-Martinez A., Gallego-Valdes M.A. (2016). Photodynamic therapy as a therapeutic alternative in vulvar lichen sclerosus: Series of 8 cases. Photodermatol. Photoimmunol. Photomed..

[46] Kandolf L., Peris K., Malvehy J., Mosterd K., Heppt M.V., Fargnoli M.C., Berking C., Arenberger P., Bylaite-Bucinskiene M., Del Marmol V. (2024). European consensus-based interdisciplinary guideline for diagnosis, treatment and prevention of actinic keratoses, epithelial UV-induced dysplasia and field cancerization on behalf of European Association of Dermato-Oncology, European Dermatology Forum, European Academy of Dermatology and Venereology and Union of Medical Specialists (Union Europeenne des Medecins Specialistes). J. Eur. Acad. Dermatol. Venereol..

[47] Morton C.A., Szeimies R.M., Basset-Seguin N., Calzavara-Pinton P., Gilaberte Y., Haedersdal M., Hofbauer G.F.L., Hunger R.E., Karrer S., Piaserico S. (2019). European Dermatology Forum guidelines on topical photodynamic therapy 2019 Part 1: Treatment delivery and established indications—Actinic keratoses, Bowen’s disease and basal cell carcinomas. J. Eur. Acad. Dermatol. Venereol..

[48] Novak B., DuBois J., Chahrour O., Papusha T., Hirt S., Philippi T., Zogel C., Osenberg K., Schmitz B., Lubbert H. (2022). Clinical Pharmacokinetics and Safety of a 10% Aminolevulinic Acid Hydrochloride Nanoemulsion Gel (BF-200 ALA) in Photodynamic Therapy of Patients Extensively Affected With Actinic Keratosis: Results of 2 Maximal Usage Pharmacokinetic Trials. Clin. Pharmacol. Drug Dev..

